# Cannabidiol Modulates the Immunophenotype and Inhibits the Activation of the Inflammasome in Human Gingival Mesenchymal Stem Cells

**DOI:** 10.3389/fphys.2016.00559

**Published:** 2016-11-24

**Authors:** Rosaliana Libro, Domenico Scionti, Francesca Diomede, Marco Marchisio, Gianpaolo Grassi, Federica Pollastro, Adriano Piattelli, Placido Bramanti, Emanuela Mazzon, Oriana Trubiani

**Affiliations:** ^1^Experimental Neurology Laboratory, IRCCS Centro Neurolesi “Bonino-Pulejo”Messina, Italy; ^2^Stem Cells and Regenerative Medicine Laboratory, Department of Medical, Oral and Biotechnological Sciences, University “G. d'Annunzio”Chieti-Pescara, Chieti, Italy; ^3^Department of Medicine and Aging Sciences, University “G. d'Annunzio”Chieti-Pescara, Chieti, Italy; ^4^Council for Research and Experimentation in Agriculture - Research Centre for Industrial Crops (CRA-CIN)Rovigo, Italy; ^5^Dipartimento di Scienze del Farmaco, Università del Piemonte OrientaleNovara, Italy

**Keywords:** cannabidiol, human gingival mesenchymal stem cells, immunophenotype, next generation sequencing, inflammasome

## Abstract

Human Gingival Mesenchymal Stem Cells (hGMSCs) are multipotential cells that can expand and differentiate in culture under specific and standardized conditions. In the present study, we have investigated whether *in vitro* pre-treatment of hGMSCs with Cannabidiol (CBD) can influence their expression profile, improving the therapeutic potential of this cell culture. Following CBD treatment (5 μM) for 24 h, gene expression analysis through Next Generation Sequencing (NGS) has revealed several genes differentially expressed between CBD-treated hGMSCs (CBD-hGMSCs) and control cells (CTR-hGMSCs) that were linked to inflammation and apoptosis. In particular, we have demonstrated that CBD treatment in hGMSCs prevented the activation of the NALP3-inflammasome pathway by suppressing the levels of NALP3, CASP1, and IL18, and in parallel, inhibited apoptosis, as demonstrated by the suppression of Bax. CBD treatment was also able to modulate the expression of the well-known mesenchymal stem cell markers (CD13, CD29, CD73, CD44, CD90, and CD166), and other surface antigens. Specifically, CBD led to the downregulation of genes codifying for antigens involved in the activation of the immune system (CD109, CD151, CD40, CD46, CD59, CD68, CD81, CD82, CD99), while it led to the upregulation of those implicated in the inhibition of the immune responses (CD47, CD55, CD276). In conclusion, the present study will provide a new simple and reproducible method for preconditioning hGMSCs with CBD, before transplantation, as an interesting strategy for improving the hGMSCs molecular phenotype, reducing the risk of immune or inflammatory reactions in the host, and in parallel, for increasing their survival and thus, their long-term therapeutic efficacy.

## Introduction

During the last decades, Mesenchymal Stem Cells (MSCs) have attracted much attention for their ability to transdifferentiate into several tissues, under specific conditions, and therefore, to potentially reconstitute the damaged organs and tissues (Zhang et al., 2009; Patel et al., 2013). They have already been isolated from different tissues, including bone marrow, adipose tissue, umbilical cord blood and oral cavity (Hass et al., 2011). In 2006, The International Society for Cellular Therapy has established the minimal criteria for defining MSCs: they must be plastic-adherent in standard culture conditions, they must express specific surface antigens like the cluster of differentiation (CD) CD105, CD73, and CD90 in flow cytometry, and they must lack expression of CD45, CD34, CD14, or CD11b, CD79a, or CD19 and HLA class II (Dominici et al., 2006). Moreover, they must show a multipotent ability to differentiate into several mesenchymal lineages such as osteoblasts, adipocytes and chondroblasts under standard *in vitro* differentiating conditions (Dominici et al., 2006).

In a recent work, our group has demonstrated the beneficial effect of the human periodontal ligament stem cells, an oral-derived MSC lineage, in experimental autoimmune encephalomyelitis models (Trubiani et al., 2016a). Among oral-derived MSCs, the human Gingival Mesenchymal Stem Cells (hGMSCs) have been recently explored since they have shared many common features to other MSCs such as a spindle-like cell morphology, plastic adherence, the expression profile of specific cell surface markers, multipotent differentiation, and even immunomodulatory functions (Xiao and Nasu, 2014; Jin et al., 2015; Fawzy El-Sayed and Dörfer, 2016). One of the major advantage of hGMSCs compared to MSCs isolated from other tissue, is their abundance and easy isolation from gingiva through a minimally invasive dental procedure (Du et al., 2016; Fawzy El-Sayed and Dörfer, 2016). Therefore, following expansion and pre-differentiation into a desired tissue type, hGMSCs potentially could be re-implanted for tissue repair in the patients.

Despite the therapeutic potential of MSCs in stem cell-based therapies, certain issues need to be addressed, such as the risk of immune rejection which can affect MSCs survival and thus their long-term fate once transplanted *in vivo*. Current approaches to prevent immune rejections are based on immunosuppressive drugs which induce an immunological tolerance in the host. However, the prolonged use of these therapies has been associated with increased risk to develop other pathological conditions, such as diabetes, hypertension, and nephrotoxicity (Merville, 2005). As a consequence, new strategies that prevent immune rejections affecting stem cell survival *in vivo* and that assure a successful long-term engraftment, are needed. Here, we propose an alternative strategy to overcome these issues which consists in preconditioning hGMSCs *in vitro* with a cannabinoid compound, the Cannabidiol (CBD). Thus far, scientists have found that cannabinoids by interacting with several receptors, including the endocannabinoid receptors type 1 and type 2 (CB1R and CB2R), can modulate several pathways, such as stem cells self-renewal, proliferation and differentiation (Galve-Roperh et al., 2013). Therefore, it is not surprising that many studies are aimed at evaluating the therapeutic potential of different exogenous cannabinoids, among which CBD has received much attention since it is devoid of psychotropic effects compared to other cannabinoids.

In this study, we have performed a Next Generation Sequencing (NGS)-based gene expression profiling in order to evaluate whether CBD treatment can modify the molecular phenotype of hGMSCs, enhancing their therapeutic efficacy.

## Materials and methods

### Extraction and isolation of CBD

Pure CBD (>99%) was isolated from Carmagnola, an Italian variety of industrial *C. sativa* and obtained from greenhouse cultivation at CREA-CIN, Rovigo (Italy), according to a standardized method of the cannabinoid purification reported by Taglialatela-Scafati et al. (2010), in order to avoid any trace of THC that could interfere in the trial or cause legal limitation. The isolation and purification of cannabinoids were done in accordance with Italian legal status (Authorization SP/106 23/05/2013 of the Ministry of Health, Rome, Italy).

### Isolation and immunophenotyping of hGMSCs

#### hGMSCs culture establishment

Gingival tissue biopsies were obtained from five healthy adult volunteers with no gingival inflammation. The gingival specimens were completely de-epithelialized with a scalpel, for the exclusion of the most of the keratinocytes resident in the gingival. In brief, the connective tissues were grinded and then washed several time with PBS (LiStarFish, Milan, Italy) and subsequently cultured using TheraPEAK™MSCGM-CD™ BulletKit serum free, chemically defined (MSCGM-CD) medium for the growth of human MSCs (Lonza, Basel, Switzerland). The medium was changed twice a week, and cells spontaneously migrating from the explant fragments after reaching about 80% of confluence, were trypsinized using Triple Select (LiStar Fish) (Diomede et al., 2014). On day 6, colonies of 50 or more cells were scored as colony-forming unit fibroblasts (CFU).

#### Cytofluorimetric evaluation

Samples were stained for surface or intracellular antigens, as previously described (Cianci et al., 2016). For surface staining, samples were resuspended in 100 μl washing buffer (0.1% sodium azide and 0.5% bovine serum albumine in PBS) containing the appropriate amount of surface antibodies; samples were incubated for 30 min at 4°C in the dark. Cells were washed (3 ml of washing buffer), centrifuged (4°C, 400 X g, 8 min), resuspended with 1ml PBS 0.5% paraformaldehyde, incubated for 5 min at RT, washed, centrifuged (4°C, 400 X g, 8min) and stored at 4°C in the dark until their acquisition. For intracellular staining, cells were resuspended in 1 ml of FACS Lysing solution (BD), vortexed and incubated at room temperature (RT) in the dark for 10 min. Samples were centrifuged (4°C, 400 X g, 8 min); 1 ml of Perm 2 (BD) was added to each tube and cells were incubated at RT in the dark for 10 min. Samples were washed and centrifuged (4°C, 400 X g, 8 min). Cells were resuspended in 100 μl of washing buffer containing the appropriate amount of intracellular antibodies and incubated for 30 min at 4°C in the dark. Cells were centrifuged (4°C, 400 X g, 8 min), resuspended with 1 ml PBS 0.5 % paraformaldehyde for 5 min at RT, washed, centrifuged (4°C, 400 X g, 8 min) and stored at 4°C in the dark until the acquisition. Cells were analyzed on a FACS Calibur flow cytometer (BD), using CellQuest™ software (BD). Quality control included regular check-ups with Rainbow Calibration Particles (BD Biosciences). Debris was excluded from the analysis by gating on morphological parameters; 20,000 non-debris events in the morphological gate were recorded for each sample. To assess non-specific fluorescence we used isotype controls. All antibodies were titrated under assay conditions and optimal photomultiplier voltages (PMT) were established for each channel. Data were analyzed using FlowJo™ software (TreeStar, Ashland, OR). Mean Fluorescence Intensity Ratio (MFI Ratio) was calculated dividing the MFI of positive events by the MFI of negative events (Trubiani et al., 2016b).

#### Multipotent differentiation of hGMSCs

##### Osteogenic differentiation

7.5 × 10^3^ cells /cm^2^ CTR-hGMSCs and CBD-hGMSCs, were seeded at 2nd passage in a 6-well cell culture plate (Falcon) and incubated overnight at 37°C, in a humidified 5% CO_2_ atmosphere. The culture medium was replaced with osteogenic induction medium represented of MSCGM-CD medium supplemented with 100 nM dexamethasone (Applichem GmbH, Darmstadt, Germany), 10 nM β-glycerol-phosphate (Applichem) and 0.05 mM 2-phosphate-ascorbic acid (Sigma-Aldrich). The medium was replaced every 3 days. At days 21 Alizarin Red S staining (Sigma-Aldrich Co.) was performed to detect calcium formation (Trubiani et al., 2015).

##### Adipogenic differentiation

CTR-hGMSCs and CBD-hGMSCs were seeded in a 6-well cell culture plate (Falcon) at a density of 2 7.5 × 10^3^ cells /cm^2^ and incubated overnight. The medium was replaced with adipogenic induction medium (Lonza) composed by induction and maintenance medium. The medium After 28 days of treatment samples were fixed and stained with Oil Red O working solution (Sigma-Aldrich) and counterstained with hematoxylin to detect the oil globules. Images were collected using light microscopy Leica DMIL (Leica Microsystem, Milan, Italy) (Trubiani et al., 2016b).

### Cell culture conditions and drug treatment

hGMSCs were cultured in monolayer using DMEM high-glucose medium (CARLO ERBA, Italy) containing 10% fetal bovine serum (FBS) (Sigma-Aldrich Co. Ltd., USA). The cells were grown in logarithmic phase at 37°C in a moisturized atmosphere of 5% CO_2_ and 95% air. For drug treatment, cells at passage 10 were grown until 70–80% confluence and then, they were treated with CBD (5 μM) for 24 h (CBD-hGMSCs). Untreated cells were used as control (CTR-hGMSCs) and cells treated with vehicle (0.1% DMSO) were also included as a control (DMSO-hGMSCs). For antagonists study, cells were incubated for 2 h with different combinations of SR141716A (CB1R antagonist; 1 μM) or AM630 (CB2R antagonist;100 nM) before CBD administration for 24 h. All antagonists were purchased from Tocris Bioscience, UK. All the experiments were made in triplicates and repeated for three independent times.

### Preparation of RNA libraries for deep sequencing

Total RNA was isolated with a Reliaprep RNA Cell Miniprep System (Promega, Milano, Italy). RNA sequencing libraries were prepared using TruSeq RNA Access library kit (Illumina, Inc., San Diego, CA, USA) according to the manufacturer's protocol. Thereafter, RNA samples (50 ng) were fragmented at 94°C for 8 min on a thermal cycler. First strand cDNA syntheses were performed at 25°C for 10 min, 42°C for 15 min, and 70°C for 15 min, using random hexameres and the SuperScript II Reverse Transcriptase (Invitrogen, Milan, Italy). In a second strand cDNA synthesis, the RNA templates were removed and a second replacement strand was generated by incorporation dUTP (in place of dTTP, to keep strand information) to generate double strands cDNA. The AMPure XP beads (Beckman Coulter) were used to clean up the blunt-ended cDNA from the second strand reaction mix. The 3′ends of the cDNA were then adenylated to facilitate the adaptor ligation in the next step. After ligation of indexing adaptors, the AMPure XP beads were used to clean up the libraries. A first Polymerase Chain Reaction (PCR) amplification step (15 cycles of 98°C for 10 s, 60°C for 30 s, and 72°C for 30 s) was performed to selectively enrich those DNA fragments that have adapter molecules on both ends and also to amplify the amount of DNA in the library. After validation of the libraries, the first hybridization step was performed using exome capture probes. Before hybridization a 2-plex pool of libraries was made, by combining 200 ng of each DNA library. The hybridization was performed by 18 cycles of 1 min incubation, starting at 94°C, and then decreasing 2°C for cycle. Then streptavidin-coated magnetic beads were used to capture probes hybridized to the target regions. The enriched libraries were then eluted from the beads and prepared for a second round of hybridization. This second hybridization (18 cycles of 1 min incubation, starting at 94°C, and then decreasing 2°C per cycle) was required to ensure high specificity of the capture regions. A second capture with streptavidin-coated beads was performed, followed by two heated wash procedures to remove the non-specific binding from the beads. The enriched libraries were then eluted from the beads and cleaned up by the AMPure XP beads, prior to a second PCR amplification. The amplification step was performed by 10 cycles (98°C for 10 s, 60°C for 30 s, and 72°C for 30 s) followed by a second PCR clean up. Finally, the libraries were quantitated by quantitative PCR using the KAPA Library Quantification Kit-Illumina/ABI Prism® (Kapa Biosystems, Inc., Wilmington, MA, USA) and validated using the Agilent High Sensitivity DNA Kit on a Bioanalyzer. The size range of the DNA fragments was measured to be in the range of 200–650 bp and peaked around 250 bp.

Libraries were normalized to 12 pM and subjected to cluster and the single read sequencing was performed for 150 cycles on a MiSeq instrument (Illumina, Inc. San Diego, CA, USA), according to the manufacturer's instructions. Finally, the libraries generated were loaded for clustering on a MiSeq Flow Cell v3 and sequenced through the MiSeq Instrument.

### Gene expression data processing

CASAVA (version 1.8.2, Illumina) software was used to demultiplex reads into specific sample and groups of the indexes. Each sample was mapped against its reference sequences “Homo sapiens UCSC hg19” using RNA-Seq Alignment version 1.0.0 with its default parameters, in particular for the Read mapping was used the TopHat 2 (Bowtie 1). The Fragments Per Kilobase Of Exon Per Million Fragments Mapped (FPKM) values were calculated for each sample using the normalized reads counts for each annotated gene [(1000 × read count) ÷ (number of gene covered bases × number of mapped fragments in million)]. Unmapped reads were removed, retaining only read pairs with both reads aligned to the reference sequences “Homo sapiens UCSC hg19.” The comparison between two different samples was visualized by a scatter plot of the LOG_2_ of the FPKM.

### Bioinformatics analysis of genes differentially expressed

Once identified the genes differentially expressed between CTR-hGMSCs and CBD-hGMSCs, the enrichment analyses of the Gene Ontology processes (GO) and the pathway analyses were conducted using the online free databases such as bioDBnet (Mudunuri et al., 2009), STRING (Szklarczyk et al., 2015) and KEEG (Kanehisa et al., 2016). This strategy, based on testing multiple tools, is recommended in order to obtain the most satisfactory results (Rhee et al., 2008). We considered only the GO categories or KEGG pathways reported to be significantly enriched.

### Immunocytochemistry

Cells on coverslips (10 mm, Thermo SCIENTIFIC, Germany) were fixed with 4% paraformaldehyde at room temperature for 20 min, followed by phosphate buffered saline (PBS, pH 7.5) washes. Then, cells were incubated with 3% hydrogen peroxide (H_2_O_2_) at room temperature for 15 min to suppress the endogenous peroxidase activity. Following three washes with PBS, cells were blocked with horse serum + 0.1% Triton X-100 for 20 min followed by incubation for overnight at 4°C with primary antibodies against examined proteins: Caspase-1 (CASP1, 1:100, Abcam), Interleukin-18 (IL-18, 1:100, Abcam), NLR family pyrin domain containing 3 (NRLP3/NALP3, 1:100, R&D Systems), Bcl2 associated X (Bax, 1:100 Santa Cruz Biotechnology Inc) and Bcl-2 (1:100 Santa Cruz Biotechnology Inc). After PBS wash, cells were incubated with biotinylated secondary antibody (1:200, Vector Laboratories, Burlingame, CA) and streptavidin ABComplex-HRP (ABC-kit from Dako, Glostrup, Denmark). The immunostaining was developed with peroxidase substrate kit DAB (Vector Laboratories, Burlingame, CA) (brown color; positive staining) and counterstaining with nuclear fast red (Vector Laboratories, Burlingame, CA) (pink background; negative staining). Microscopy was performed using light microscopy (LEICA DM 2000 combined with LEICA ICC50 HD camera). Immunocytochemistry images were assessed for densitometry analysis using LEICA Application Suite V4.2.0 software to calculate the percentage of positive staining of the cells. All images are representative of three independent experiments.

### Protein extraction and Western blot analysis

In order to extract proteins, cells were washed with ice-cold PBS and lysed using Buffer A [320 mM Sucrose, 10 mM, 1 mM EGTA, 2 mM EDTA, 5 mM NaN3, 50 mM NaF, β-mercaptoethanol, and protease/phosphatase inhibitor cocktail (Roche, USA)] in ice for 15 min, followed by centrifugation at 1000 g for 10 min at 4°C. The supernatant was served as cytosolic extract. The pellet was further lysed using Buffer B [150 mM NaCl, 10 mM Tris-HCl (pH 7.4), 1 mM EGTA, 1 mM EDTA, Triton X-100, and protease/phosphatase inhibitor cocktail (Roche, USA)] in ice for 15 min, followed by centrifugation at 15,000 g for 30 min at 4°C. The supernatant was collected and used as nuclear extract. Then, the concentrations of proteins were assessed by using the Bio-Rad Protein Assay (Bio-Rad, Segrate, Italy) using BSA as standard, and 30 μg of total extracts from each sample were analyzed. Total proteins were separated on sodium dodecyl sulfate-polyacrylamide mini-gels and then transferred onto polyvinylidene fluoride membranes (Immobilon-P Transfer membrane, Millipore), blocked with PBS containing 5% nonfat dried milk (PM) for 45 min at room temperature, and subsequently incubated at 4°C overnight with a specific antibody for the cannabinoid receptor 1 (CB1R, 1:200; ThermoFisher, Scientific), for caspase-1 (CASP1, 1:1000, Abcam) and for pro-caspase-1 (pro-CASP1, 1:1000, Abcam).

The membranes were incubated with secondary antibodies, HRP-conjugated goat anti-mouse IgG or HRP-conjugated goat anti-rabbit IgG (1:2000; Santa Cruz Biotechnology, Inc.) for 1 h at room temperature. To ascertain that blots were loaded with equal amounts of protein lysates, they were also incubated with antibody for GAPDH HRP Conjugated (1:1000; Cell Signaling Technology). The relative expression of protein bands was visualized using an enhanced chemiluminescence system (Luminata Western HRP Substrates, Millipore) and protein bands were acquired and quantified with ChemiDoc™ MP System (Bio-Rad) and analyzed by using the software Image J.

### Statistical data analysis

The statistical analyses to determine the proportion of differentially expressed genes between two samples on the read counts were performed with the Cufflinks Assembly & DE package version 2.0.

Immunocytochemical and Western blot data were analyzed by using the software GraphPad Prism 6.0 (GraphPad Software, La Jolla, CA) and applying the one-way ANOVA statistic test, followed by a Bonferroni *post-hoc* test for multiple comparisons. A *p*-value of < 0.05 was considered statistically significant.

## Results

### Cytofluorimetric characterization of hGMSCs

**Figure 1:**
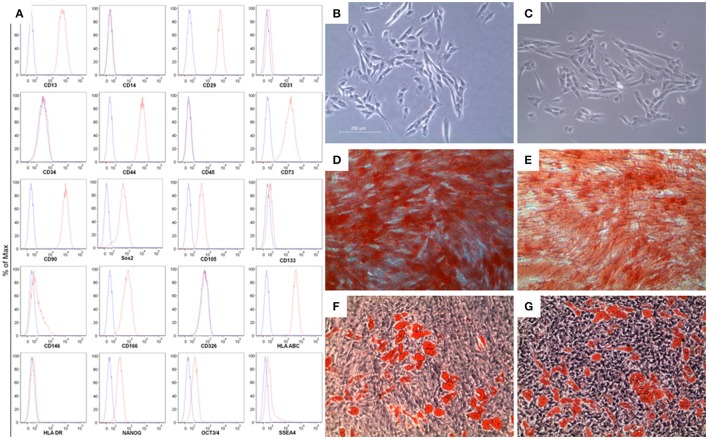
**Characterization of gingiva-derived mesenchymal stem cells (hGMSCs). (A)** Flow cytometry phenotype of hGMSCs at the second passage during *in vitro* cultures of surface (CD13, CD14, CD29, CD31, CD34, CD44, CD45, CD73, CD90, CD105, CD117, CD133, CD146, CD166, CD326, HLA-ABC, and HLA-DR) and intracellular (SSEA4, Oct3/4, Sox2, and NANOG) marker expression levels were detected. Red histograms show the distribution of each antigen expression, whereas Blue histograms represent the distribution of the respective background control. **(B)** The gingival connective tissue-derived mesenchymal stem cells showed colony-forming potency and plastic-adherent characteristics at day 6. **(C)** CBD-hGMSCs and CTR-hGMSCs showed spindle-shaped, fibroblast-like morphology under light microscope. **(D)** Osteogenic differentiation at day 21 after Alizarin Red S staining highlighted calcium deposits in CTR-hGMSCs and in **(E)** CBD-hGMSCs. **(F)** CTR-hGMSCs and **(G)** CBD-hGMSCs induced to a dipogenic differentiation after 28 days showed many oil droplets at cytoplasmic level stained with Oil Red O solution. Mag: 10X; bar: 200 μm.

Flow cytometry was used to assess the MSC immunoprofile. In Figure 1, section A, we have shown that hGMSCs expressed surface molecules such as CD13, CD29, CD44, CD73, CD90, CD105, CD 146, CD166, and the human leukocyte antigen (HLA)-ABC, while they were negative for the subsequent markers: CD14, CD31, CD34, CD45, CD117, CD133, CD326, HLA-DR, according to the minimal criteria for defining MSCs (Dominici et al., 2006). Moreover, hGMSCs expressed pluripotency associated markers like OCT3/4, NANOG, SOX2, SSEA4.

### Morphological and functional characterization of hGMSCs

The first CFU appeared after 6 days of the initiation of the primary culture. Under optical microscopy, primary cells showed spindle-shaped with fibroblastic-like morphology (Figure 1B). No differences in morphological features have been evidenced after CBD treatment for 24 h (Figure 1C). Moreover, we have proved the multipotent potential of hGMSCs to differentiate in osteoblasts and adipocytes. During osteogenic differentiation, at day 21, after Alizarin Red S staining, mineralized extracellular deposits were observed in CTR-hGMSCs and in CBD-hGMSCs (Figures 1D,E). For adipogenic differentiation, Oil Red O solution stained oil globules containing many lipid-rich vacuoles at cytoplasmic level in CTR-hGMSCs and in CBD-hGMSCs 28 days of culture in adipogenic medium (Figures 1E,F).

### Global gene expression

To explore the effect of CBD treatment on the expression profile of the hGMSCs, we performed a high-throughput sequencing through the NGS platform. This technique allowed us to map about 14.320 for the CTR-hGMSCs group and 10.158 for CBD-hGMSCs group. Statistical analysis revealed 5843 genes differentially expressed following CBD treatment, among which 2798 were down-regulated, while 3045 were up-regulated.

### CBD treatment modulated the expression of genes related to inflammation, immune response and apoptosis

Interestingly, here we found that CBD totally inhibited the expression of 25 genes in CBD-hGMSCs (False Discovery Rate (FDR), *q*-value < 0.05). According to GO processes, these suppressed genes belonging to the following GO categories: apoptotic process and regulation of apoptotic process (H1FX, CASP6, PDCL3, SIVA1, ING4, AQP1, MIEN1, CAPN3,CASP1, WWOX, IFI6, GAS1, PTN, PPP3CC, CARD16, PYCARD), inflammatory response (FLT3LG, DDT, NLRP3, BRCC36, LY96, ATAT1, PLGRKT) and innate immune response (HLA-H) and signal transduction (GNG10). In Table 1A have been reported the GO process categories, the expression levels of CTR-hGMSCs and CBD-hGMSCs (Exp_Value), the Fold Change (FC) and the False Discovery Rate (FDR) for each aforementioned genes.

**Table 1A:** **CBD treatment suppressed genes linked to apoptosis, inflammatory and innate immune responses**.

**Gene**	**Description**	**Go processes**	**CTR-hGMSCs Exp_Value**	**CBD-hGMSCs Exp_Value**	**LOG_10_FC**	**FDR Q_VALUE**
AQP1	Aquaporin 1	Apoptotic process, regulation of apoptotic process extrinsic apoptotic signaling pathway	6.01	0.00	−5.78	9.79e-05
CAPN3	Calpain 3	Apoptotic process, regulation of apoptotic process, inflammatory response	5.16	0.00	−5.71	9.79e-05
CARD16	Caspase recruitment domain family member 16	Regulation of apoptotic process, inflammatory response	6.60	0.00	−5.82	9.79e-05
CASP1	Caspase 1	Apoptotic process, regulation of apoptotic process, inflammatory response	8.22	0.00	−5.92	9.79e-05
CASP6	Caspase 6	Apoptotic process, regulation of apoptotic process,	2.19	0.00	−5.3	9.79e-05
GAS1	Growth arrest specific 1	Regulation of apoptotic process, regulation of response to stimulus	14.63	0.00	−6.17	2.96e-02
H1FX	H1 histone family member X	Apoptotic DNA fragmentation, nucleosome assembly	6.41	0.00	−5.81	9.79e-05
IFI6	Interferon alpha inducible protein 6	Apoptotic process, innate immune response	5.07	0.00	−5.71	9.79e-05
ING4	Inhibitor of growth family member 4	Apoptotic process, regulation of apoptotic process	5.88	0.00	−5.77	9.79e-05
MIEN1	Migration and invasion enhancer 1	Apoptotic process, regulation of apoptotic process, cell redox homeostasis	16.44	0.00	−6.22	9.79e-05
PDCL3	Phosducin like 3	Apoptotic process, protein folding	11.78	0.00	−6.07	9.79e-05
PPP3CC	Protein phosphatase 3 catalytic subunit gamma	Apoptotic process, positive regulation of apoptotic process	8.82	0.00	−5.95	9.79e-05
PTN	Pleiotrophin	Positive regulation of apoptotic process	6.62	0.00	−5.82	9.79e-05
PYCARD	PYD and CARD domain containing	Regulation of apoptotic process, inflammatory response, innate immune response	8.12	0.00	−5.91	9.79e-05
SIVA1	SIVA1 apoptosis inducing factor	Apoptotic process, regulation of apoptotic process	21.16	0.00	−6.33	9.79e-05
WWOX	WW domain containing oxidoreductase	Apoptotic process, regulation of apoptotic process	8.16	0.00	−5.91	2.14e-02
FLT3LG	fms related tyrosine kinase 3 ligand	Regulation of apoptotic process, inflammatory response	11.22	0.00	−6.05	9.79e-05
DDT	D-dopachrome tautomerase	Inflammatory response	16.71	0.00	−6.22	9.79e-05
NLRP3	NLR family, pyrin domain containing 3	Inflammatory response, apoptotic process	3.17	0.00	−5.34	9.79e-05
BRCC36	BRCA1/BRCA2-containing complex subunit 3	Inflammatory response, G2 DNA damage checkpoint	5.02	0.00	−5.70	9.79e-05
LY96	Lymphocyte antigen 96	Inflammatory response, innate immune response, regulation of apoptotic process	14.95	0.00	−6.17	9.79e-05
ATAT1	Alpha tubulin acetyltransferase 1	Inflammatory response	4.88	0.00	−5.69	9.79e-05
PLGRKT	Plasminogen receptor with a C-terminal lysine	Inflammatory response	5.68	0.00	−5.75	9.79e-05
HLA-H	Major histocompatibility complex, class I, H	Antigen processing and presentation, regulation of immune response	8.08	0.00	−5.91	9.79e-05
GNG10	G protein subunit gamma 10	G-protein coupled receptor signaling pathway, signal transduction	6.37	0.00	−5.80	9.79e-05

*The differential expression between CBD-hGMSCs and CTR-hGMSCs is given in Fold Change (log_10_ FC). GO processes indicate their classification in different biological processes. Instead, the statistical significance is indicated by False Discovery Rate (FDR), p-values ≤ 0.05 were considered statistically significant*.

However, by interrogating the KEGG database for pathway analysis, we found only a pathway predicted to be statistically significant for this class of genes: the NOD-like receptor signaling (FDR = 0.006; Table 1B), an intracellular platform for the assembly of protein complexes known as inflammasomes, which includes NLRP3, CASP1, and PYCARD (Table 1A) (Shaw et al., 2011). NALP3 is a NOD-like receptor and together with the adaptor protein PYCARD forms a CASP1 activating complex, in response to a dangerous stimulus, known as the NALP3 inflammasome, which is the best characterized inflammasome complex (Schroder and Tschopp, 2010). Active CASP1 can eventually process IL-1 and IL-18 precursors, enhancing multiple pro-inflammatory pathways, including NF-κB, the mitogen-activated protein kinase (MAPK) and Interferon (IFN) (Fuentes-Antrás et al., 2014). Our gene expression results indicated a downregulation of the genes implicated in the NALP3-inflammasone pathway in CBD-HGMSCs.

**Table 1B:** **CBD suppressed the expression of genes belonging to the NOD-like receptor signaling pathway**.

**KEGG pathway**	**Gene symbol**	**FDR**
NOD-like receptor signaling pathway	NLRP3, CASP1, PYCARD	0.006

Although for the other genes suppressed by CBD and reported in Table 1A, none statistical pathways were predicted by the KEEG software, these preliminary results have encouraged us to further investigate the potential of CBD to modulate genes implicated in immune response, inflammation and apoptosis.

### CBD treatment attenuated the expression of pro-inflammatory genes

Gene expression profiling has showed that CBD treatment in hGMSCs downregulated the expression of genes codifying for pro-inflammatory cytokines (IL6ST, IL-1β, and IL-18), Interleukin receptors or subunits (IL1R1, IL11RA, IL13RA), the Toll-like receptor adaptor (MYD88), Interferon Gamma Receptors (IFNGR1 and IFNGR2), Mitogen-Activated Protein Kinases (MAPK1, MAPK12, and MAPK14), transcription factors (STAT3 and STAT6), the complex of NFκB (NFKB2, NFKB3/RELA), and the Matrix Metallopeptidase 3 (MMP3) (Table 2A). Specifically, these genes can be classified in the following GO terms: response to cytokine, regulation of immune response, regulation of defense response. etc. (Table 2A). The GO annotations, the expression levels (Exp-Value), the fold changes (FC) and the False Discovery Rates (FDR) of these genes differentially expressed between CBD-hGMSCs and CTR-hGMSCs have been reported in Table 2A.

**Table 2A:** **CBD treatment reduced the expression of pro-inflammatory genes**.

**Gene**	**Description**	**Go processes**	**CTR-hGMSCs Exp_Value**	**CBD-hGMSCs Exp_Value**	**LOG_10_ FC**	**FDR Q_VALUE**
CARD8	Caspase Recruitment Domain Family Member 8	Regulation of inflammatory response	2.87	0.91	−0.49	3.52e-04
CTSB	Cathepsin B	Response to cytokine, regulation of inflammatory response, regulation of defense response	150.13	104.14	−0.15	9.79e-05
HSP90AA1	Heat shock protein 90 alpha family class A member 1	Regulation of apoptotic process, negative regulation of apoptotic process, regulation of apoptotic signaling pathway	423.83	724.1	0.23	9.79E-05
IFNGR1	Interferon Gamma Receptor 1	Response to cytokine, regulation of immune response, regulation of defense response	14.43	7.32	−0.29	9.79e-05
IFNGR2	Interferon Gamma Receptor 2	Response to cytokine, regulation of immune response, regulation of defense response	40.42	35.86	−0.05	4.5e-02
IL11RA	Interleukin 11 Receptor Subunit Alpha	Response to cytokine	23.77	12.22	−0.28	9.79e-05
IL13RA1	Interleukin 13 Receptor Subunit Alpha 1	Response to cytokine, inflammatory response, cell surface receptor signaling pathway,	6.01	0.60	−0.99	9.79e-05
IL1B	Interleukin 1 Beta	Response to cytokine, inflammatory response, regulation of inflammatory response	6.48	4.71	−0.13	1.91 e-03
IL1R1	IL1R1 Interleukin 1 Receptor Type 1	Response to cytokine, regulation of defense response, regulation of inflammatory response	6.48	4.71	−0.13	1.90e-03
IL6ST	Interleukin 6	Response to cytokine, regulation of immune response, regulation of defense response, regulation of inflammatory response	24.86	22.91	−0.03	0.021783
IL18	Interleukin 18	Response to cytokine, inflammatory response, regulation of inflammatory response	0.89	−1.06	−0.31	2.58e-05
MAPK1	Mitogen-Activated Protein Kinase 1	Response to cytokine, inflammatory response, regulation of inflammatory response, MAPK cascade	35.18	27.59	−0.10	2.82e-03
MAPK12	Mitogen-Activated Protein Kinase 12	Inflammatory response, regulation of inflammatory response, MAPK cascade	7.16	4.35	−0.21	8.23e-03
MAPK14	Mitogen-Activated Protein Kinase 14	Inflammatory response, regulation of immune response, regulation of defense response	14.06	5.83	−0.38	9.79e-05
MMP3	Matrix Metallopeptidase 3	Inflammatory response, regulation of inflammatory response, extracellular matrix disassembly	85.78	55.05	−0.19	9.79e-05
MYD88	Myeloid Differentiation Primary Response Gene 88	Response to cytokine, regulation of immune response, regulation of defense response, regulation of inflammatory response	12.55	2.62	−0.67	9.79e-05
NFKB2	Nuclear Factor Kappa B Subunit 2	Response to cytokine, regulation of immune response	8.64	5.53	−0.19	4.50e-05
NFKBIA	NFKB Inhibitor Alpha	Response to cytokine, inflammatory response, negative regulation of NF-kB transcription factor activity	28.48	34.80	0.08	1.34e-02
RELA	RELA Proto-Oncogene, NF-κB Subunit	Response to cytokine, regulation of immune response, regulation of defense response	17.21	11.29	−0.18	9.79e-05
STAT3	Signal Transducer And Activator Of Transcription 3	Response to cytokine, inflammatory response, regulation of inflammatory response	45.10	20.27	−0.34	9.79e-05
STAT6	Signal Transducer And Activator Of Transcription 6	Response to cytokine, regulation of immune response, inflammatory response, regulation of inflammatory response	47.66	37.66	−0.10	9.79e-05

*Gene expression levels of CTR-hGMSCs and CBD-hGMSCs are given in log_10_, as well as the Fold Changes (FC). GO processes indicate their classification in different biological processes. Instead, the statistical significance is indicated by the False Discovery Rate (FDR), p-values ≤ 0.05 were considered statistically significant*.

According to KEEG pathway predictions, this group of genes can be clustered in the most following significant pathways: the NOD-like receptor signaling pathway, the TNF signaling pathway, the Jak-STAT signaling pathway and the NF-kB signaling pathway (Table 2B). These results altogether indicated that CBD negatively regulates differential pathways involved in inflammatory responses, but in particular the NOD-like receptor signaling pathway.

**Table 2B:** **CBD negatively modulated the transcription of genes involved in NOD-like receptor signaling pathway and other inflammatory pathways**.

**KEGG pathway**	**Gene symbol**	**FDR**
NOD-like receptor signaling pathway	CARD8, IL1B, IL18, MAPK1, MAPK12, MAPK14, NFKBIA, RELA	2.3e-14
TNF signaling pathway	MMP3, IL1B, MAPK1, MAPK12, MAPK14, NFKBIA, RELA	1.43e-09
Jak-STAT signaling pathway	STAT6, IFNGR1, IFNGR2, IL13RA1, IL6ST, STAT3	1.43e-08
NF-kB signaling pathway	MYD88, IL1B, IL1R1, NFKBIA, NFKB2, RELA	2.19e-08

*The statistical significance is indicated by FDR, p-values of ≤ 0.05 were considered statistically significant*.

### CBD prevented the activation of the NALP3-inflammasome in hGMSCs

**Figure 2:**
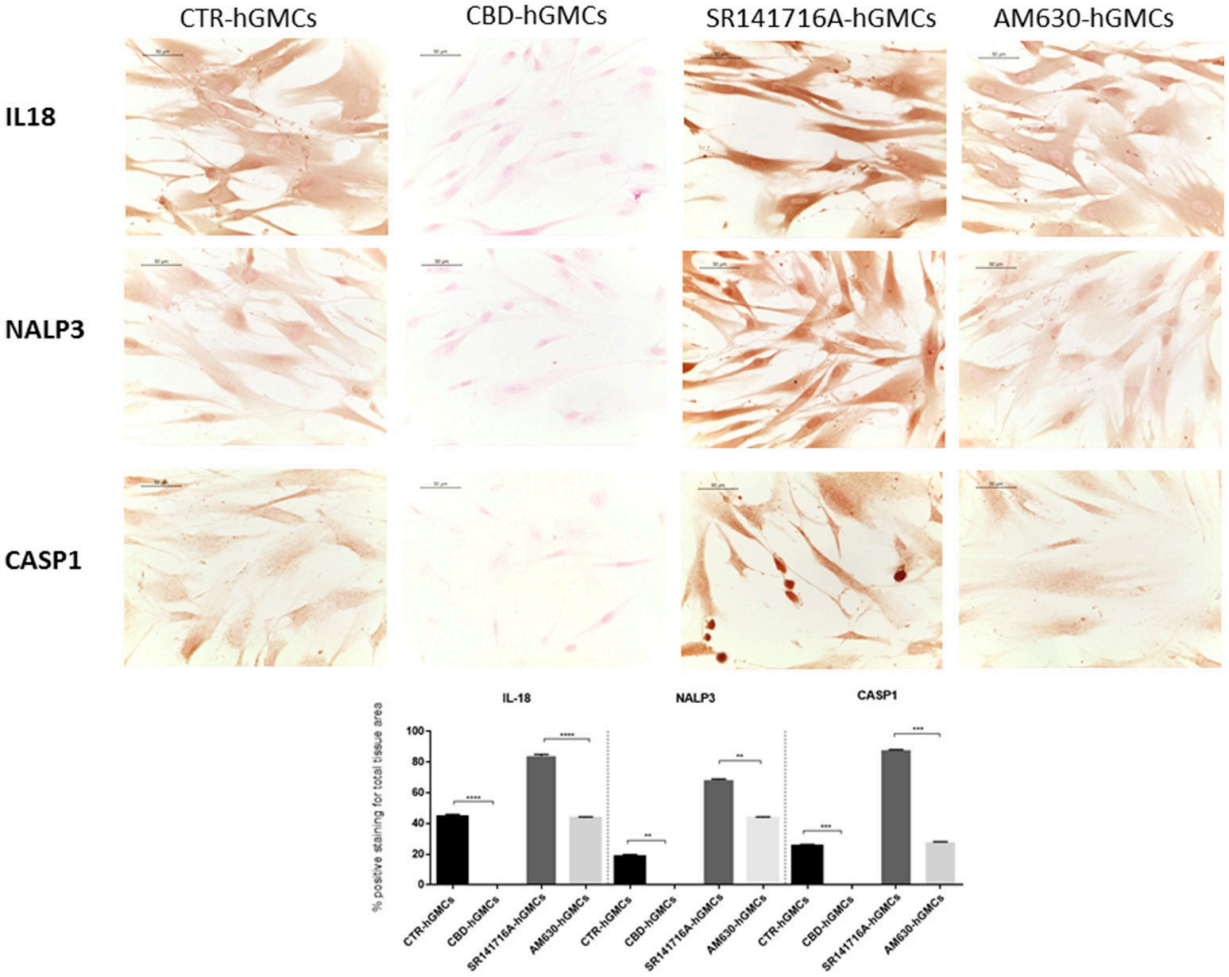
**Immunocytochemical staining for IL18, NALP3 and CASP1**. CBD-hGMSCs showed a negative staining for IL-18, NALP3, and CASP1 compared to CTR-hGMSCs. Instead, hGMSCs-SR141716A and hGMSCs-AM630 showed a significant positive staining for IL-18, NALP3, and CASP1 compared to CBD-hGMSCs. The graph represented the densytometric quantitative analysis. For IL-18 CTR-hGMSCs vs. CBD-hGMSCs ^***^p < 0.001; SR141716A-hGMSCs vs. AM630-hGMSCs ^****^p < 0.0001. For NALP3 CTR-hGMSCs vs. CBD-hGMSCs ^**^p < 0.01; SR141716A-hGMSCs vs. AM630-hGMSCs ^**^p < 0.01. For CASP-1 CTR-hGMSCs vs. CBD-hGMSCs ^***^p < 0.001; SR141716A-hGMSCs vs. AM630-hGMSCs ^***^p < 0.001.

**Figure 3:**
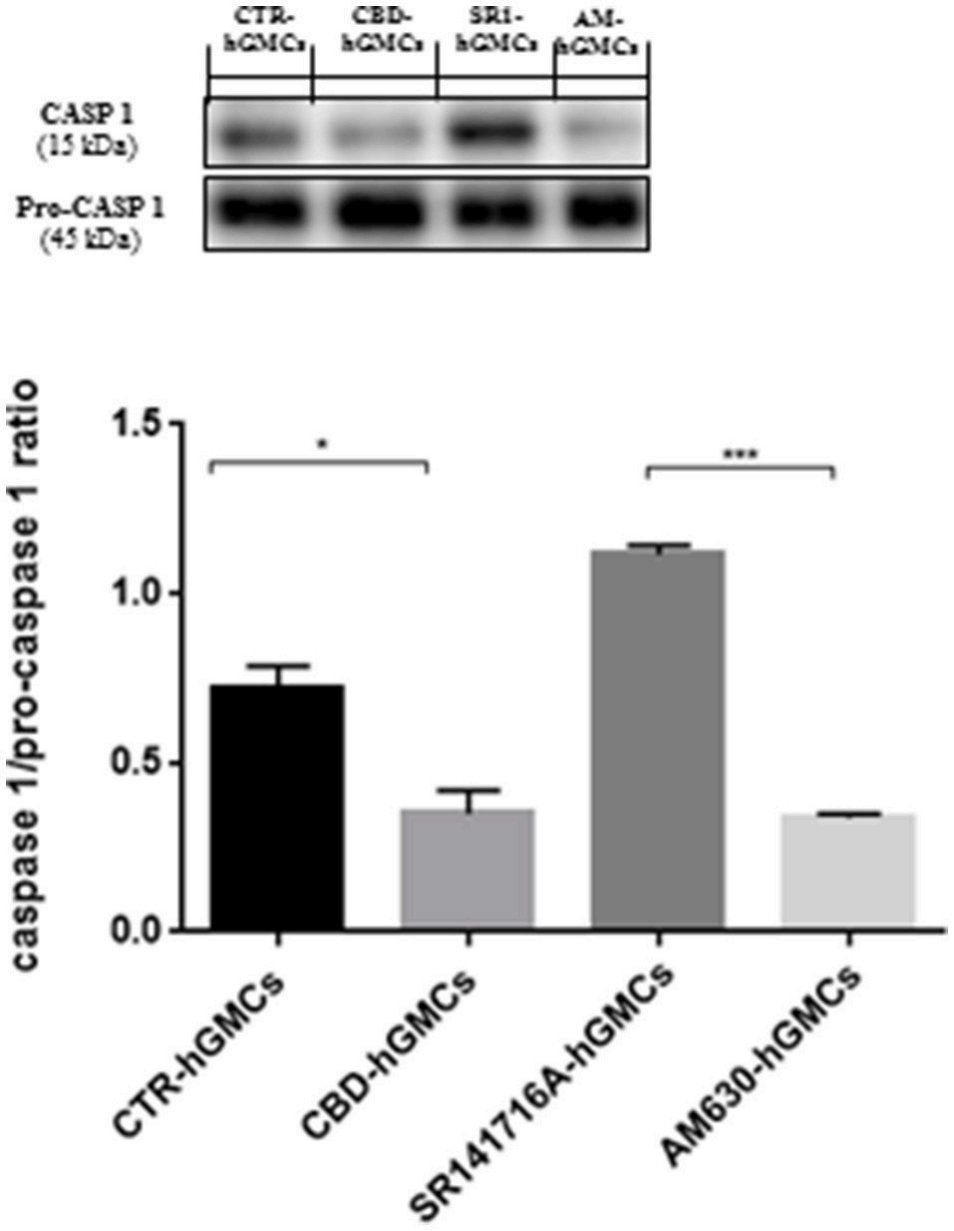
**Western blot analysis for CASP1**. CASP1 expression is decreased in CBD-hGMSCs compared to CTR-DMSO). The levels of CASP1 are significant increase in SR141716A-hGMSCs compared with CBD-hGMSCs. CTR-hGMSCs vs. CBD-hGMSCs ^*^p < 0.05; SR141716A-hGMSCs vs. AM630-hGMSCs ^***^p < 0.001.

The NALP3-inflammasome has been correlated with several inflammatory conditions and increasing evidences have indicated that the inhibition of the inflammasome could be a promising strategy for treating inflammatory disorders and also for preventing allograft-rejections in cell-based therapies (Foley, 2013; Coll et al., 2015; Shah et al., 2015). Our gene expression data have revealed a downregulation of several genes codifying for key players of the NALP3-inflammasome in CBD-hGMSCs. In order to confirm the ability of CBD to modulate the NALP3-inflammasome, we have performed immunocytochemistry and western blot analyses. Achieved immune-localization data have shown that NALP3, IL-18, and CASP1 were completely negative in CBD-hGMSCs, in a significant manner compared to CTR-hGMSCs (Figure 2). A representative negative control of immunocytochemistry is showed in Supplementary Material. The expression levels of CASP1 were also investigated by Western blotting and normalized on pro-CASP1, showing a reduced CASP1 expression in CBD-hGMSCs compared to CTR-hGMSCs (Figure 3).

Moreover, in order to understand whether the inhibition of the inflammasome by CBD could be mediated by the cannabinoid receptors (CB1R and CB2R), we have pre-treated hGMSCs with selective receptor antagonists for CB1R (SR141716A) and CB2R (AM630), before CBD treatment for 24 h. Immunochemistry has showed that SR141716A reverted completely the downregulation of NALP3, IL-18, and CASP1 induced by CBD, instead AM630 was also able to antagonize only partially the CBD effects. Western blotting has confirmed that blocking CB1R reverted the CBD-mediated reduction of CASP1 (Figure 3).

**Figure 4:**
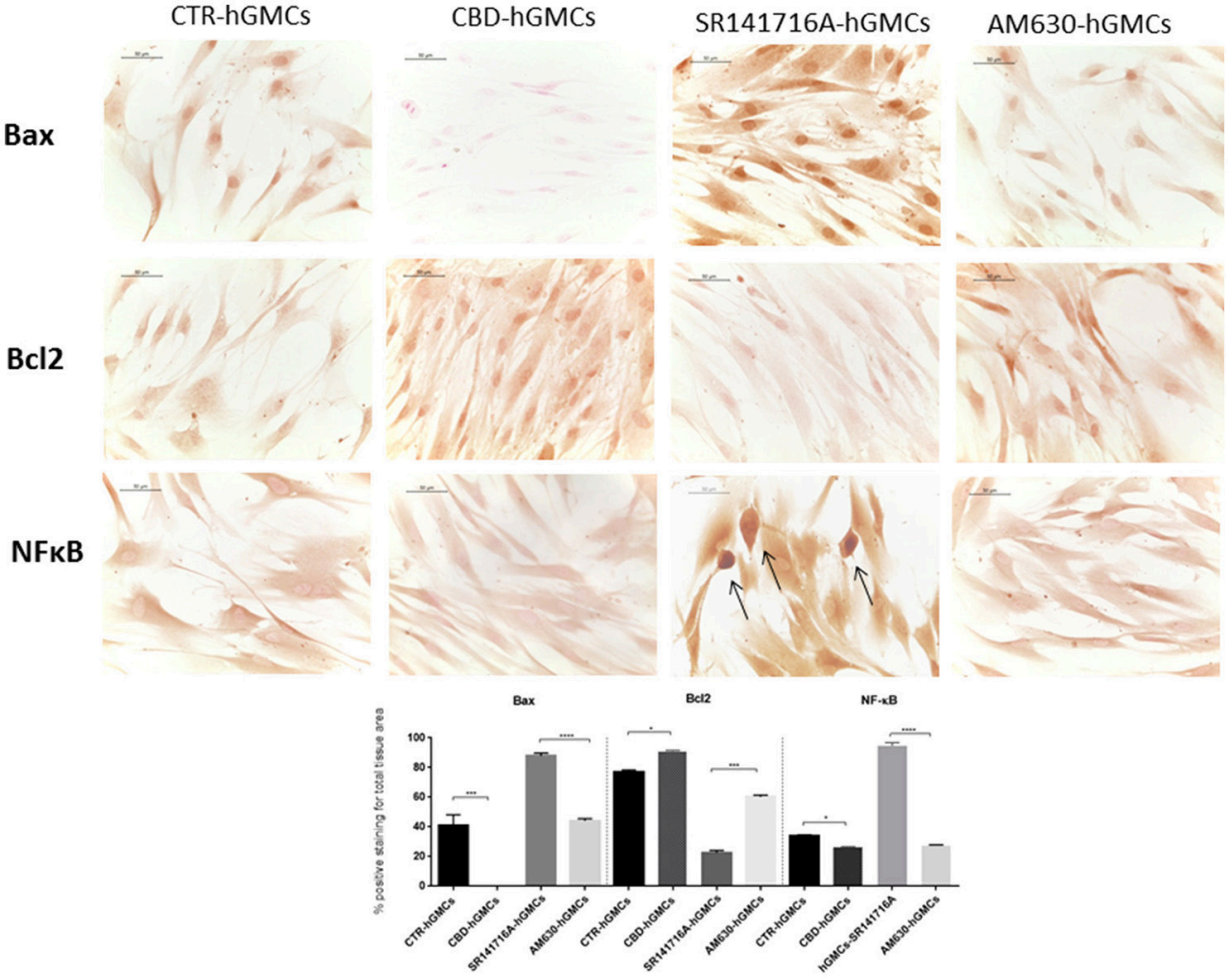
**Immunocytochemical staining for Bax, Bcl2 and NFκB**. CBD-hGMSCs showed a negative staining for Bax, a reduced expression for NF-KB and a positive staining for Bcl2, compared to CTR-hGMSCs. hGMSCs-SR141716A showed a marked positive cytoplasmatic/nuclear staining for NF-kB, as indicated by the arrows. Moreover, hGMSCs-SR141716A showed an increased nuclear expression for Bax and reduced expression for Bcl2 at both cytoplasmatic and nuclear compartment, compared to CBD-hGMSCs. Instead, hGMSCs-AM630 showed a reduced expression for Bax and Bcl2 compared to CBD-hGMSCs. Whereas, no differences statistically differences were found for the NF-kB expression between CBD-hGMSCs and hGMSCs-AM630. The graph represented the densytometric quantitative analysis. For Bax CTR-hGMSCs vs. CBD-hGMSCs ^***^p < 0.001; SR141716A-hGMSCs vs. AM630-hGMSCs ^****^p < 0.0001. For Bcl2 CTR-hGMSCs vs. CBD-hGMSCs ^*^p < 0.05; SR141716A-hGMSCs vs. AM630-hGMSCs ^***^p < 0.001. For NF-kB CTR-hGMSCs vs. CBD-hGMSCs ^*^p < 0.05; SR141716A-hGMSCs vs. AM630-hGMSCs ^****^p < 0.0001.

Moreover, we evaluated the expression of NF-κB since it is considered as a regulator of NALP3 activity (Bauernfeind et al., 2009), finding that NF-κB levels were statically reduced by CBD treatment, as demonstrated by immunocytochemistry analyses (Figure 4). These results suggested that CBD promotes the inactive state of the NALP3-inflammasome pathway.

**Figure 5:**
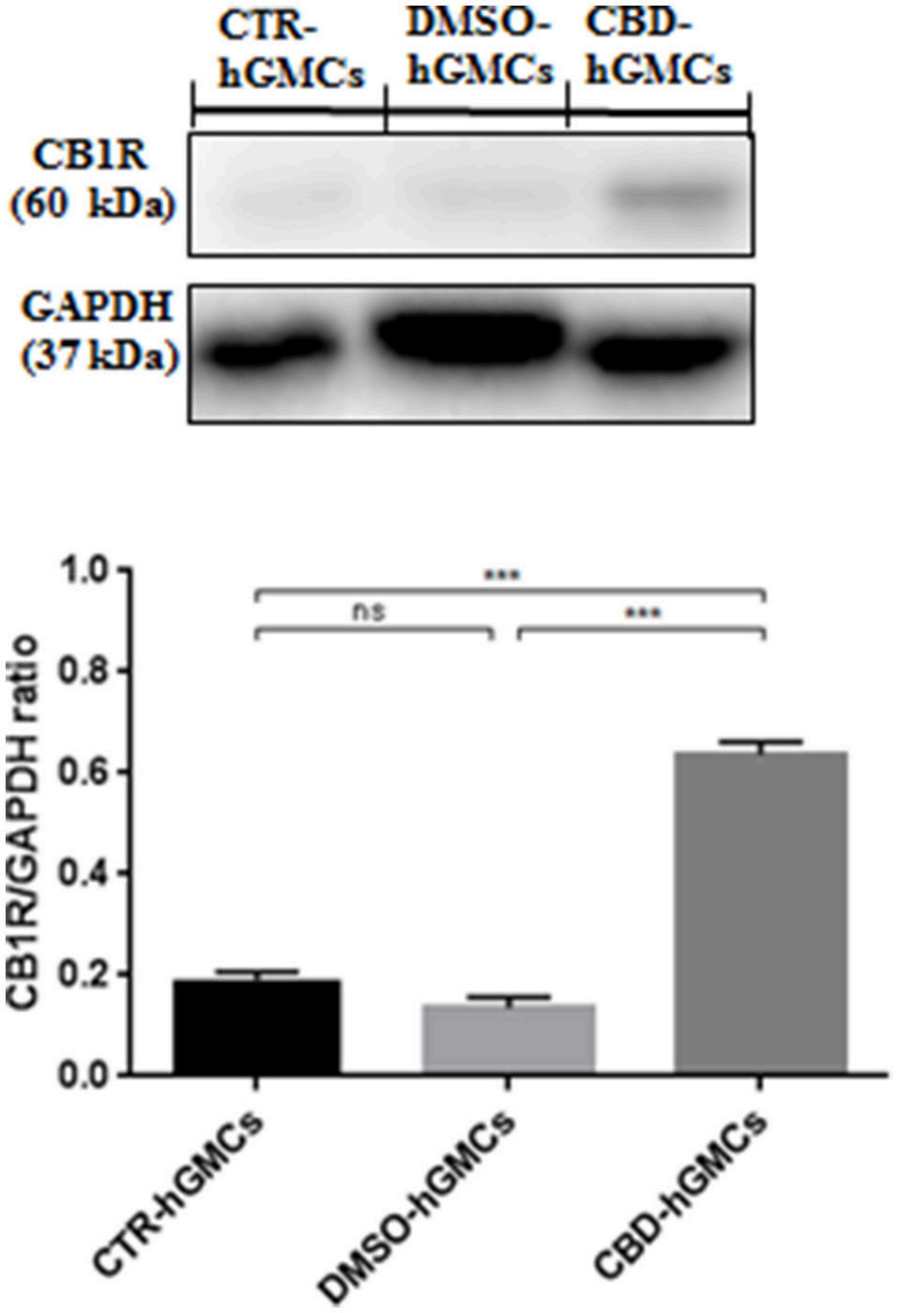
**Western blot analysis for CB1R**. CB1R expression is increased in CBD-hGMSCs compared to control groups (DMSO-hGMSCs and CTR-hGMSCs). ^***^p < 0.001. ns, no statistical differences.

**Figure 6:**
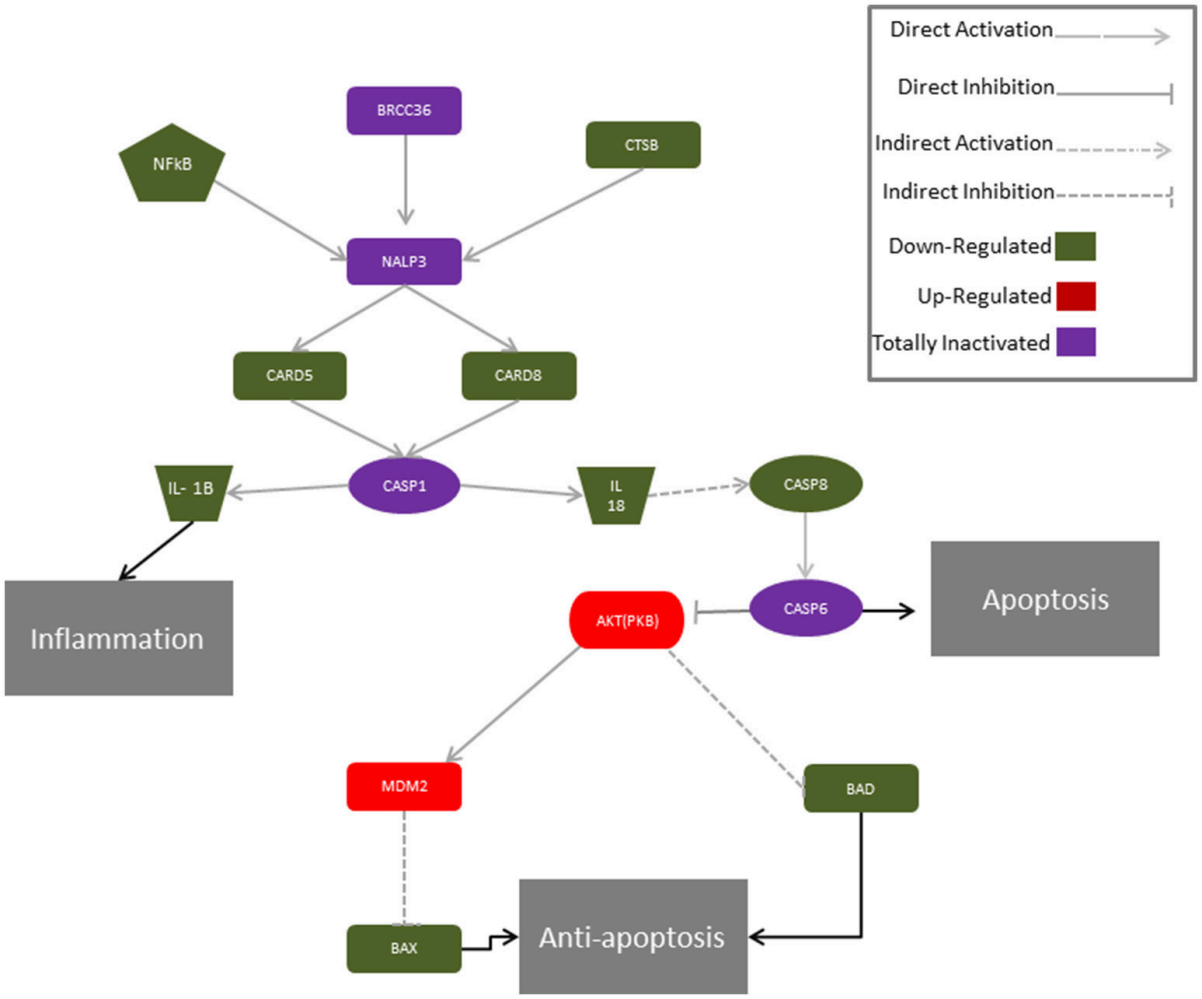
**Proposed molecular mechanism for CBD modulation of the genes of the NALP3-inflammasome in hGMSCs**. The activation of the NALP3-inflammasome is regulated by NF-kB which promotes NALP3 deubiquitination via activation of BRCC36. NALP3 interacts with CASP1 through CARD5 and CARD8. Activated CASP1 cleaves pro-IL-1 beta and pro-IL-18, leading to subsequent release of mature cytokines which trigger inflammation. In parallel, IL-18 indirectly via CASP8 and CASP6 can stimulate apoptosis. In hGMSCs, BRCC6, NALP3 and CASP1 transcripts are suppressed (violet) and the expression of the downstream mediators (CARD5, CARD8, IL1β, IL18, CASP8) is downregulated (green), while CASP8 is totally suppressed (violet). Instead, the pro-survival AKT1 and MDM2 were upregulated (red), leading to the downregulation of BAX and BAD (green).

Moreover, only the CB1 agonist has shown to antagonize the CBD-mediated reduction of NF-kB, indicating that CBD may act in a CB1R dependent manner, while CB2R is likely to be not involved in this signaling. These results might suggest that CBD can interact with both CB1R and CB2R, although it acts preferably via a CB1R mechanism, showing a selectivity for the CB1R respect to the CB2R in hGMSCs. Additionally, Western blot results (Figure 5) have showed an increased CB1R expression in CBD-hGMSCs compared to control groups (DMSO-hGMSCs and CTR-hGMSCs). A schematic representation of the NALP3-inflammasome pathway in CBD-hGMSCs is illustrated in the Figure 6.

### CBD reduced the expression of pro-apoptotic genes

Following infusion of MSCs, the hostile environment could induce apoptosis few hours after transplantation. Thus, researchers had tried to develop new strategies to improve survival of MSCs, such as preconditioning of MSCs by cultivation under peroxide hydrogen, hypoxic and serum deprivation (Farzaneh Taban et al., 2016).

Here, we have previously showed that CBD treatment in hGMSCs inhibited the expression of several genes correlated to the apoptosis, according to the GO biological processes. In this study, we have also found that CBD downregulated the expression of additional genes related to apoptosis, among which we detected genes codifying for the TNF receptor superfamily members (TNFRSF10B, TNFRSF11B, TNFRSF12A, and TNFRSF19), the initiator caspases (CASP4 and CASP8), the pro-apoptotic mediators (BAX, BAD, BID, BCL7B, BCL2L13, CYCS), and the apoptotic peptidase APAF-1 (Table 3A). Some of these genes were predicted by KEGG to be involved in the apoptotic pathway in a significant manner, as reported in Table 3B.

**Table 3A:** **CBD treatment reduced the expression of pro-apoptotic genes**.

**Gene**	**Description**	**Go processes**	**CTR-hGMSCs Exp_Value**	**CBD-hGMSCs Exp_Value**	**LOG_10_FC**	**FDR Q_VALUE**
AKT1	AKT serine/threonine kinase 1	Regulation of apoptotic process, negative regulation of apoptotic process, regulation of apoptotic signaling pathway	47.16	59.52	0.10	9.79e-05
APAF1	Apoptotic peptidase activating factor 1	Regulation of apoptotic process, positive regulation of cysteine-type endopeptidase activity involved in apoptotic process, regulation of apoptotic signaling pathway	7.14	5.34	−0.12	2.77e-03
BAD	BCL2 associated agonist of cell death	Positive regulation of cysteine-type endopeptidase activity involved in apoptotic process, regulation of apoptotic signaling pathway	12.89	11.18	−0.06	4.34e-05
BAX	BCL2 associated X, apoptosis regulator	Regulation of apoptotic process, extrinsic apoptotic signaling pathway	37.48	13.20	−0.45	9.79e-05
BCL2L13	BCL2 like 13	Regulation of apoptotic process, positive regulation of cysteine-type endopeptidase activity involved in apoptotic process	9.96	4.33	−0.36	9.79e-05
BCL7B	BCL tumor suppressor 7B	Regulation of apoptotic process, regulation of apoptotic signaling pathway	8.40	1.58	−0.72	9.79e-05
BID	BH3 interacting domain death agonist	Positive regulation of cysteine-type endopeptidase activity involved in apoptotic process, regulation of apoptotic process, extrinsic apoptotic signaling pathway	5.33	1.37	−0.60	9.79e-05
CASP4	Caspase 4	Regulation of apoptotic process, negative regulation of apoptotic process, regulation of apoptotic signaling pathway	74.8	23.66	−0.50	9.79e-05
CASP8	Caspase 8	Positive regulation of cysteine-type endopeptidase activity involved in apoptotic process, regulation of apoptotic process, extrinsic apoptotic signaling pathway	10.07	3.79	−0.42	9.79e-05
CYCS	Cytochrome c, somatic	Positive regulation of cysteine-type endopeptidase activity involved in apoptotic process, negative regulation of apoptotic process, regulation of apoptotic signaling pathway	9.07	3.42	−0.42	9.79e-05
HSP90AA1	Heat shock protein 90 alpha family class A member 1	Regulation of apoptotic process, negative regulation of apoptotic process, regulation of apoptotic signaling pathway	423.83	724.1	0.23	9.79e-05
MDM2	MDM2 proto-oncogene	Regulation of apoptotic process, negative regulation of apoptotic process, regulation of apoptotic signaling pathway	15.7341	17.81	0.05	9.728e-02
PIK3CA	Phosphatidylinositol-4,5-bisphosphate 3-kinase catalytic subunit alpha	Regulation of apoptotic process, negative regulation of apoptotic process, regulation of apoptotic signaling pathway	14.77	18.38	0.095	1.35e-03
PIK3CB	Phosphatidylinositol-4,5-bisphosphate 3-kinase catalytic subunit beta	Regulation of apoptotic process, negative regulation of apoptotic process, regulation of apoptotic signaling pathway	3.33	5.60	0.22	9.79e-05
TNFRSF10B	TNF receptor superfamily member 10B	Positive regulation of cysteine-type endopeptidase activity involved in apoptotic process, regulation of apoptotic process, extrinsic apoptotic signaling pathway	63.87	38.70	−0.21	9.79e-05
TNFRSF11B	TNF receptor superfamily member 11B	Regulation of apoptotic process, regulation of apoptotic signaling pathway	136.65	97.87	−0.14	9.79e-05
TNFRSF12A	TNF receptor superfamily member 12A	Regulation of apoptotic process, extrinsic apoptotic signaling pathway	8.54	2.97	−0.47	9.07e-3
TNFRSF19	TNF receptor superfamily member 12A	Regulation of apoptotic process, regulation of apoptotic signaling pathway	25.96	9.10	−0.41	9.79e-05

*Both the expression levels (Exp_Value) and the fold changes (FC) are expressed in log_10_. GO terms indicate their classification in different biological processes. Instead, the statistical significance is indicated by False Discovery Rate (FDR), p-values ≤ 0.05 were considered statistically significant*.

**Table 3B:** **CBD modulated the apoptotic pathway in hGMSCs**.

**KEGG pathway**	**Gene symbol**	**FDR**
Apoptosis	APAF-1, AKT-1, BAX, BAD, BID, CYCS, CASP8, MDM2, PIK3CA PIK3CB, TNFRSF10B	4.01e-17

*The statistical significance is indicated by the False Discovery Rate (zFDR), p-values ≤ 0.05 were considered statistically significant*.

In parallel, CBD enhanced the transcription of the phosphoinositid-3 kinase (PIK3) subunits (PIK3CA and PIK3CB) and AKT1 serine/threonine kinase 1 (AKT1). The upregulation of the PI3K/Akt signaling has been associated with increased stem cell survival (Hossini et al., 2016).

Moreover, we have observed a negative staining for the pro-apoptotic mediator Bax in CBD-hGMSCs and positive staining for the anti-apoptotic protein Bcl-2, by immunocytochemistry. These data confirmed that CBD treatment inhibited apoptosis in hGMSCs (Figure 4). By pre-treating cells with the cannabinoid receptor agonists, we found that this inhibition seems to be mainly mediated by the CB1R (Figure 4). Our results suggested that CBD treatment may improve stem cell survival through two complementary strategies: by reducing the expression of pro-apoptotic mediators and by promoting the anti-apoptotic ones.

### CBD modified the immunophenotype of hGMSCs

MSCs have been reported to exert immunosuppressive properties in the host (Schu et al., 2012). However, evidence also suggests that they can also elicit a allogeneic immune response (Nauta et al., 2006). Thus, we investigated whether CBD was able to modulate the cell surface expression of immunogenic markers. We detected a differential expression of the well-known mesenchymal markers: specifically CD13, CD29, and CD73 were upregulated in CBD-hGMSCs (Table 4), while CD44, CD90, and CD166 were downregulated (Table 4).

**Table 4:** **Mesenchymal markers differentially expressed between CTR-hGMSCs and CBD-hGMSCs**.

**Gene**	**Description**	**Go processes**	**CBD-HGMSCS Exp_Value**	**CBD-HGMSCS Exp_Value**	**LOG_10_FC**	**FDR Q_VALUE**
CD13 (ANPEP)	Alanyl Aminopeptidase	Single organismal cell-cell adhesion	114.68	120.90	0.02	4.36e-02
CD29 (ITGB1)	Integrin Subunit Beta 1	Cell adhesion Cell-matrix adhesion Single organismal cell-cell adhesion	511.60	666.69	0.11	9.79e-05
CD73 (NT5E)	Nt5e 5'-Nucleotidase Ecto	Cell adhesion Single organismal cell-cell adhesion	314.08	360.84	0.06	9.79e-05
CD44	CD44 Molecule	Cell adhesion Cell-matrix adhesion Single organismal cell-cell adhesion	306.34	295.16	−0.01	2.96e-02
CD90 (THY1)	Thy-1 Cell Surface Antigen	Cell adhesion Cell-matrix adhesion Single organismal cell-cell adhesion	75.20	58.37	−0.11	9.79e-05
CD166 (ALCAM)	Activated Leukocyte Cell Adhesion Molecule	Cell adhesion, single organismal cell-cell adhesion	58.64	53.18	−0.04	1.30e-02

*The expression levels (Exp_Value) and the fold changes (FC) are expressed in log_10_. The statistical significance is indicated by the False Discovery Rate (FDR) value, p-values ≤ 0.05 were considered statistically significant*.

Furthermore, we found that following CBD treatment, other genes codifying for surface antigens were differentially expressed, in particular those involved in the inhibition of the immune system activation such as CD47, CD55, and CD276 were upregulated whereas, the expression levels of the costimulatory molecules CD40, CD46, CD81, and CD82, as well as the levels of the T-cells and macrophage activators CD59, CD68, and CD99 and, the immune surface markers CD109 and CD151 were decreased by CBD (Table 5). The GO process categories, the expression levels (Exp_Value), the fold change (FC) and the False Discovery Rate (FDR) have been given in Table 5. None pathway statistically significant was found for this group of genes, according to KEEG database.

**Table 5:** **CBD modulated the transcription of genes codifying for the antigenic repertoire of hGMSCs**.

**Gene**	**Description**	**Go processes**	**CBD-HGMSCS Exp_Value**	**CBD-HGMSCS Exp_Value**	**LOG_10_FC**	**FDR Q_VALUE**
CD276	CD276 molecule	Regulation of immune system process, negative regulation of T cell proliferation, negative regulation of inflammatory response	20.86	25.95	0.09	3.64e-4
CD47	CD47 molecule	Regulation of immune system process, leukocyte migration, integrin-mediated signaling pathway	13.11	16.64	0.10	2.77e-03
CD55	CD55 molecule	Regulation of immune system process, Complement and coagulation cascades	12.29	15.22	0.09	0.45e-02
CD40	CD40 molecule	Immune system process, immune response, regulation of immune system process	5.60	1.73	−0.50	9.79e-05
CD46	CD46 molecule	Regulation of immune system process, T cell mediated immunity	54.35	42.68	−0.10	9.79e-05
CD59	CD59 molecule	Immune system process, immune response, regulation of immune system process	130.87	78.57	−0.22	9.79e-05
CD68	CD68 molecule	Immune system process	134.37	116.70	−0.06	9.79e-05
CD81	CD81 molecule	Regulation of immune system process, cell surface receptor signaling pathway	404.95	270.42	−0.17	9.79e-05
CD82	CD82 molecule	Cell surface receptor signaling pathway, regulation of immune system process	24.53	15.88	−0.18	9.79e-05
CD99	CD99 molecule	Immune system process, cell adhesion	685.80	638.84	−0.03	3.64e-03

*Both the expression levels (Exp_Value) and the fold changes (FC) are expressed in log_10_. GO processes indicate their classification in different biological processes. Instead, the statistical significance is indicated by False Discovery Rate (FDR), p-values ≤ 0.05 were considered statistically significant*.

## Discussion

The ability of hGMSCs to self-renew, to differentiate into different tissues, together with their easy accessibility and abundance, renders them a promising alternative source of MSCs for regenerative medicine. However, prior to their clinical applications, further investigations are required to ensure that they do not elicit immune or inflammatory responses that could affect their efficacy and survival *in vivo*.

In this work, we asked whether preconditioning donor cells (hGMSCs) with CBD, before transplantation in the host, could modify their gene expression profile, improving their therapeutic efficacy.

Global gene expression data have showed that CBD modulated the expression of 5843 genes in hGMSCs, among which, we found that CBD treatment downregulated the expression of genes correlated to inflammation and apoptosis. Specifically, we have detected a reduced expression of several key genes involved in the NOD-like receptor signaling and more specifically, belonging to the NALP3 inflammasome complex. The NALP3-inflammasome could be considered as an integrated signaling system which converges multiple signaling pathways such as cell death and innate immune responses (Nakanishi et al., 2001). Indeed, following a dangerous stimulus, NALP3 binds its adaptor protein PYCARD to assembly the inflammasome complex which recruits CASP1 (Latz et al., 2013). CASP1 in turn promotes the cleavage of the pro-inflammatory cytokines such as IL-1β and IL-18 (Lalor et al., 2011), which promote the inflammatory and immune responses (Nakanishi et al., 2001; Netea et al., 2010). Here, we have found that CBD promotes the inactive state of the NALP3-inflammasome pathway in hGMSCs by inhibiting some key members of this signaling, including NALP3, CASP1, and IL18, *via* a preferably CB1R mechanism, which does not exclude completely the CB2R involvement. Moreover, we have found that CBD led to increased CB1R expression in hGMSCs. These findings are in according with previous studies which have showed an increased CB1R expression following *in vitro* exposure to exogenous or endogenous cannabinoids (Börner et al., 2007; Laprairie et al., 2013).

Additionally, we have observed a reduced expression of NF-κB, a crucial transcription factor which promotes the transcription of several pro-inflammatory mediators, including NALP3, both at transcriptional and protein levels. Moreover, the blockade of CB1R, through the administration of a specific antagonist, led to increased NF-kB expression, while no statistically changes were observed after the exposure to CB2R antagonist, indicating a direct involvement of CB1R in this signaling.

Overall, our results have suggested that CBD negatively regulated the NALP3-inflammasome activation, mainly via a CB1R mechanism. Although a direct modulation of the inflammasome complex by the cannabinoid receptors CB1R and CB2R, has been already investigated by previous studies, it role remains still controversial. Indeed, it has been reported that CB2R activation, by inhibiting the NLRP3-inflammasome pathway, played a protective role in experimental models of inflammatory and autoimmune disorders (Shao et al., 2014; Ke et al., 2016). Conversely, Jourdan et al. (2013) have showed that CB1R mediated the NALP3-inflammasome activation and, thus inflammation in macrophages derived from rat model of diabetes.

However, accumulating evidences have highlighted the NALP3 inflammasome as potential target for treating inflammation and for preventing graft failures (Foley, 2013; Jankovic et al., 2013; Coll et al., 2015), thus we proposed that CBD treatment, by antagonizing the NALP3 inflammasome activation in hGMSCs, renders them less predispose to trigger inflammation, thus more resistant to the hostile microenvironment, once transplanted in the host. All these evidences suggested that preconditioning hGMSCs with CBD might be a strategy to improve cell engraftment in the context of stem cell-based therapy.

Following MSCs infusion, the hostile environment could induce apoptosis few hours after transplantation, as consequence, new strategies to counteract apoptosis or to improve survival of MSCs are needed. In this study, we have showed that CBD pre-treatment in hGMSCs correlated with the downregulation of several genes involved in apoptosis. Moreover, we have demonstrated that Bax expression was totally suppressed in CBD-hGMSCs, while Bcl2 levels were increased, confirming an anti-anti-apoptotic role for CBD in these cells. We have also found that this pro-survival effect of CBD could be mediated by the CB1R. These results are corroborated by other studies which have showing that CB1R activation correlated with reduced apoptosis (Gómez del Pulgar et al., 2000) and increased mesenchymal stem cell survival (Gowran et al., 2013).

Immune rejection is one of the major complication that may occur with stem cell transplantation. The main targets for the immune response to transplanted grafts are major histocompatibility complex (MHC)-encoded molecules (Ayala García et al., 2012). Despite, the hGMSCs have showed to express the human leukocyte antigen (HLA) major histocompatibility complex (MHC) class I and to be negative for MHC II (Castro-Manrreza et al., 2015), they have still not been adequately characterized with respect to their immunogenic properties. Several studies reported that CBD could have a role in the inhibition of the graft rejection T-cells mediated (Kaplan et al., 2008; Nagarkatti et al., 2010; Robinson et al., 2013), exerting immunosuppressive properties.

Here, we have found that CBD treatment modified the expression of genes codifying for surface antigens in hGMSCs. Specifically, in CBD-hGMSCs we detected upregulated levels of the well-known MSCs markers CD13, CD29, CD73, CD13, CD29, CD73, involved in promoting MSCs adhesion and migration. The overexpression of these markers has been associated with improved MSCs therapeutic efficacy (Ode et al., 2011; Rahman et al., 2014). Moreover, CBD treatment led to the down-regulation of the MSCs markers CD44, CD90, CD166. Interestingly, the downregulation of CD90, CD44, and CD166 has been linked to a more differentiated stem cell phenotype and to a less tumorigenic potential (Moraes et al., 2016). Additionally, we detected others surface markers involved the activation of the immune system which expression was downregulated by CBD such as CD109, CD151, CD40, CD46, CD59, CD68, CD81, CD82, CD99. CD109, and CD151 are surface markers expressed by different cell types of the immune system (Zhang et al., 2015; Seu et al., 2016). CD59 and CD99 and play a role in of T-cells activation and migration (Kimberley et al., 2007), whereas CD68 promotes macrophage activation (Ji, 2012). CD40, CD46, CD81, and CD82 are immune-costimulatory antigens which promote the activation of different cells of the immune system (Levy et al., 1998; Shibagaki et al., 1998; Zaffran et al., 2001; Elgueta et al., 2009; Johnson and Ruffell, 2009; Saleh et al., 2013). In parallel, CBD treatment led to the upregulation of antigens which negatively regulate T cell activation via the disruption of APC/T interactions as well as of antigen processing and presentation, such as CD47, CD55, and CD276. In particular, CD47 is expressed on most innate immune cells able to dampen the inflammatory immune response (Latour et al., 2001). Instead, CD276 (B7-H3) is a negative regulator of T cell proliferation, as well as CD55 (DAF), a complement inhibitor protein that suppresses T cells activation *in vivo*. Of note, transgenic overexpression of human DAF is a strategy to reduce the immune rejection complement-mediated in xenotransplants (Liu et al., 2005). Based on our results, we suggested that CBD treatment by modifying the molecular immunophenotype of hGMSCs, make them less immunogenic. A less immunogenic phenotype can potentially prevent the allogenic immune responses without requiring a systemic immune suppression in the host.

In conclusion, we found that CBD negatively regulated the NALP3-inflammasome activation and cell death, *via* the inhibition of apoptosis, through a CB1R-dependent. Moreover, CBD treatment reduced the immunogenicity of hGMSCs.

In the present study, we will provide a new simple and reproducible method for preconditioning hGMSCs with CBD before transplantation. This method could represent an interesting strategy for reducing the risk of immune reactions following cell transplantation, and in parallel, for increasing their survival and thus, their long-term therapeutic efficacy.

## Ethics statement

The protocol and informed consent from human gingival biopsies were carried out in accordance with the approved guidelines of Medical Ethics Committee at the Medical School, “G. d'Annunzio” University, Chieti, Italy (n°266/17.04.14).

## Author contributions

RL wrote the manuscript. DS provided technical support for NGS experiments and data analysis. FD and MM were involved in acquisition, analysis and interpretation of data and helped in revising the manuscript critically for important intellectual content. GG and FP have performed CBD isolation and purification. AP has performed the recruitment of patients for hGMSCs explanation. PB, EM, and OT conceived and designed the experiments, and were involved in revising the manuscript.

## Funding

This study was supported by current research funds 2016 of Health Ministry to IRCCS Centro Neurolesi “Bonino-Pulejo.”

### Conflict of interest statement

The authors declare that the research was conducted in the absence of any commercial or financial relationships that could be construed as a potential conflict of interest.
