# Supplementary figures and images for: Cannabidiol Modulates the Immunophenotype and Inhibits the Activation of the Inflammasome in Human Gingival Mesenchymal Stem Cells
Source: Front Physiol. 2016 Nov 24;7:559. doi: 10.3389/fphys.2016.00559 (PMC5121123; doi:10.3389/fphys.2016.00559)

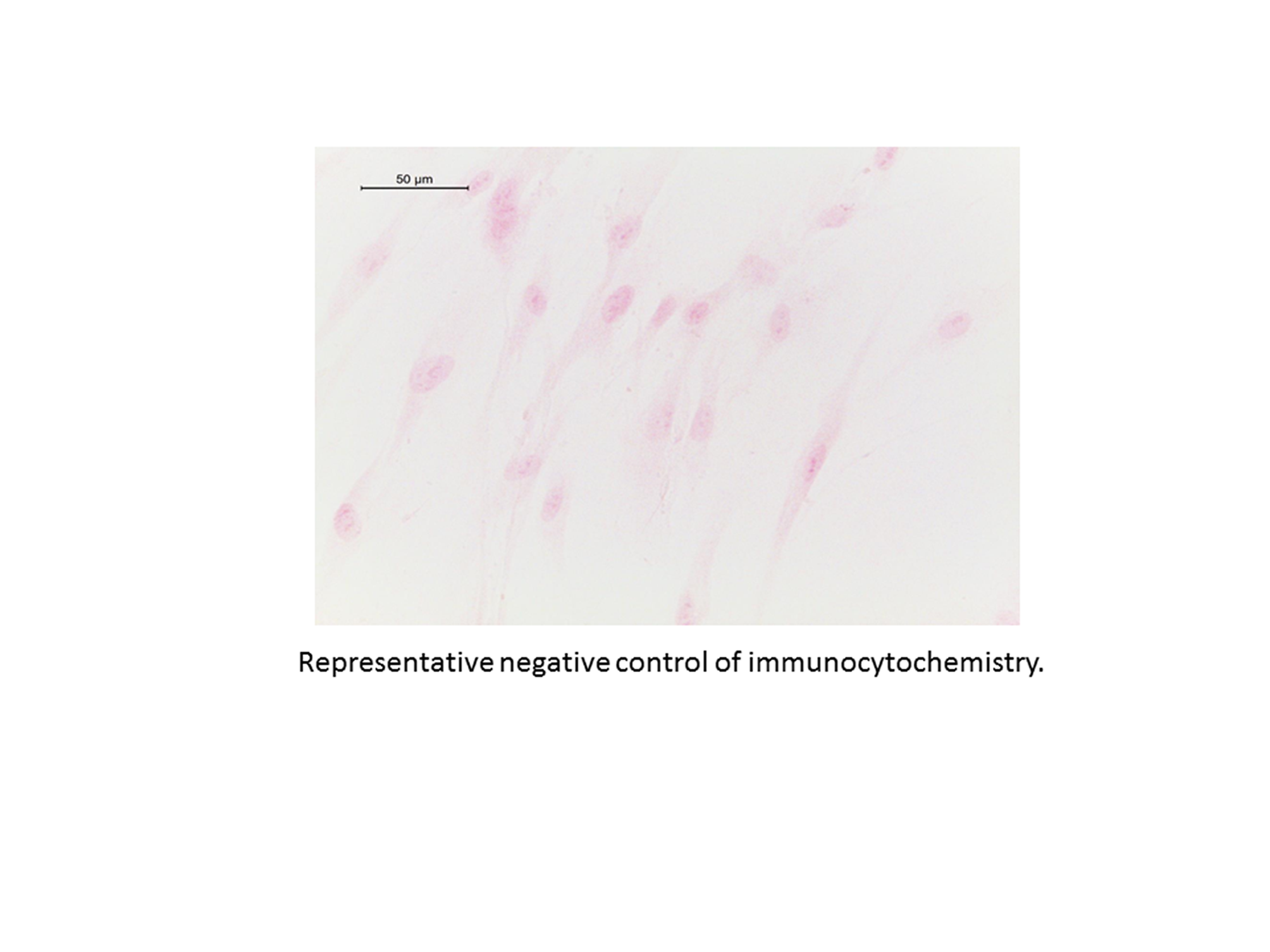

Supplement: Supplementary file 1 [file Image1.TIF]
